# Command Electro-Optical Switching of Photoaligned Liquid Crystal on Photopatterned Graphene

**DOI:** 10.1038/s41598-017-11903-9

**Published:** 2017-09-18

**Authors:** Andrii Varanytsia, Liang-Chy Chien

**Affiliations:** Liquid Crystal Institute, Kent State University, 1425 Lefton Esplanade, Kent, Ohio, 44242 USA

## Abstract

We report command electro-optical switching on photolithographically-patterned graphene into a high-density electrode pattern for a high-transmission in-plane-switching (IPS) liquid crystal device. A highly-effective liquid crystal photoalignment method is used to maximize the field-driven optical contrast of a prototyped device. A non-contact and low-temperature photoalignment allows delicate surface treatment required for successful processing of graphene layer into an IPS electrode structure. Electro-optic performance of the graphene-based single pixel laboratory IPS prototype demonstrates the application potential of graphene for liquid crystal electro-optic devices with complex and high-definition electrode patterns.

## Introduction

Development of transparent electronic and electro-optical (EO) devices is a topic of major interest in modern science and technology. Good electrical conductivity, excellent optical transmittance and mechanical flexibility generate continuous interest in graphene as a candidate transparent conductive material for applications in a large variety of optoelectronic and photonic devices including solar cells, light emitting devices, photodetectors, smart windows, and liquid crystal devices (LCD)^1–4^. Large Young’s modulus and elasticity of atomically thin graphene^5^ and graphene oxide^6^ sheets enable extraordinary flexibility^7^ and consideration of these materials for flexible applications with large deformations^8^.

Graphene-based electronic devices have stimulated a continuous interest for application of graphene as a transparent conductive electrode in LC EO devices^9–14^. Consideration of graphene and graphene oxide transparent electrodes has been extended for applications in voltage controlled liquid crystal (LC) terahertz phase shifters^15–18^, polymer dispersed liquid crystal (PDLC) devices on rigid glass and flexible plastic substrates^19,20^, and flexible plastic contact lenses^21^. These works have demonstrated suitability of transparent graphene electrodes for application in LC EO devices, and at the same time have indicated technical challenges related to mechanical processing and handling of graphene. Nearly all LC EO devices require accurate control of the average orientation of the LC molecules, known as LC director, which is typically defined by the alignment layer deposited above electrically conducting electrodes. Traditionally, the alignment layer is a thin polymeric film which is mechanically rubbed in order to define the direction of desired LC orientation^22,23^. Being an atomically thin carbon film, graphene by its nature is much more vulnerable to irreversible mechanical damage which may be caused during the rubbing process compared to much thicker and mechanically more robust films of solid inorganic transparent conductive oxides. Additionally, graphene can be delaminated from the substrate as result of a contact with n-methyl-2-pyrrolidone (NMP) organic solvent which is a major component of all solutions of commercial alignment layer materials such as polyimides (PI)^13,15,17^. A partial solution of the alignment issue for LC on graphene electrodes could be to use a water soluble poly(vinyl alcohol) (PVA) as alignment layer^10–12^. However, challenges to align the LC using rubbing of either PVA or PI alignment layers require to consider non-contact alignment for the best optical contrast ratio (CR) and EO performance. Such techniques are photoalignment in the case of planar alignment and polymer stabilization for vertical alignment (VA)^14,24–26^.

The photoalignment layer is a thin film of a photosensitive material capable of creating a high-quality orientational ordering of LC director on its surface. The orientation direction of LC director is defined by irradiation treatment with linearly polarized light having a narrow wavelength spectrum and accurately controlled polarization axis with respect to the surface of the substrate^27,28^. The photoalignment is a well-known technique for LC alignment via an anisotropic surface interaction capable of a large area uniform alignment for display applications^29–31^. At the same time, photoalignment has a unique potential for creation of complex LC orientation patterns implementing lattices of topological features for active control of soft matter LC systems^32^. Until now an azo dye LC photoalignment has only been demonstrated on a non-patterned porous graphene layer for terahertz LC phase shifter^16^.

In this work we report in details on development of the fabrication process capable of producing a high-resolution patterns of interdigitated electrodes for modern in-plane switching (IPS)^33,34^, “fishbone” VA^25,26^, or fringe field switching (FFS)^35,36^ LCD modes. We produce a high-quality graphene-based single pixel laboratory LCD prototype using commercially available photoalignment material. Direct comparison of prototypes with identical design demonstrates better optical transmittance and equivalent EO performance of IPS device with graphene electrodes compared to reference device with ITO electrodes.

## Experimental Results and Discussion

**Figure 1:**
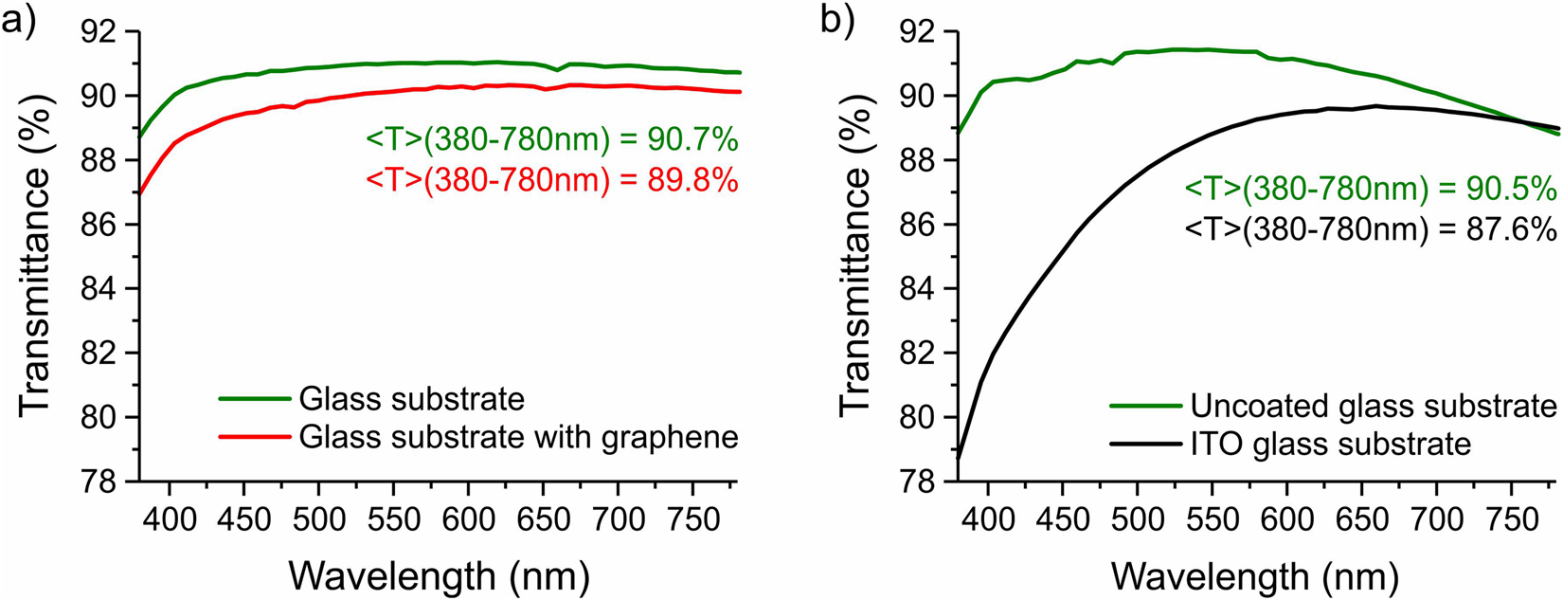
Transmission spectra of uncoated glass substrates and glass substrates coated with transparent conductors: (**a**) single layer graphene, (**b**) ITO.

Graphene films were grown on Cu films using a chemical vapor deposition (CVD) process and transferred onto a quartz glass substrates using a thin PMMA film as the supporting substrate^37,38^. A single layer graphene film absorbs from 0.6% (at 780 nm) to 1.8% (at 380 nm) of incident light, as shown on Fig. 1(a). An average transmittance of 0.6 mm thick quartz glass substrate used in our experiments in the range of wavelength from 380 nm to 780 nm is 90.7%, and is 89.8% when the same substrate coated with graphene film. In comparison, an average transmittance of a standard 1.1 mm thick uncoated soda-lime glass is 90.5%, and 87.6% for the same type of substrate commercially coated with the ITO film. Transmission spectra of graphene and reference ITO substrates are normalized to the transmittance of air. The shorter wavelength drop of ITO transmittance would be slightly improved by the refractive index matching with the LC mixture in a filled ITO cell^13^.

**Figure 2:**
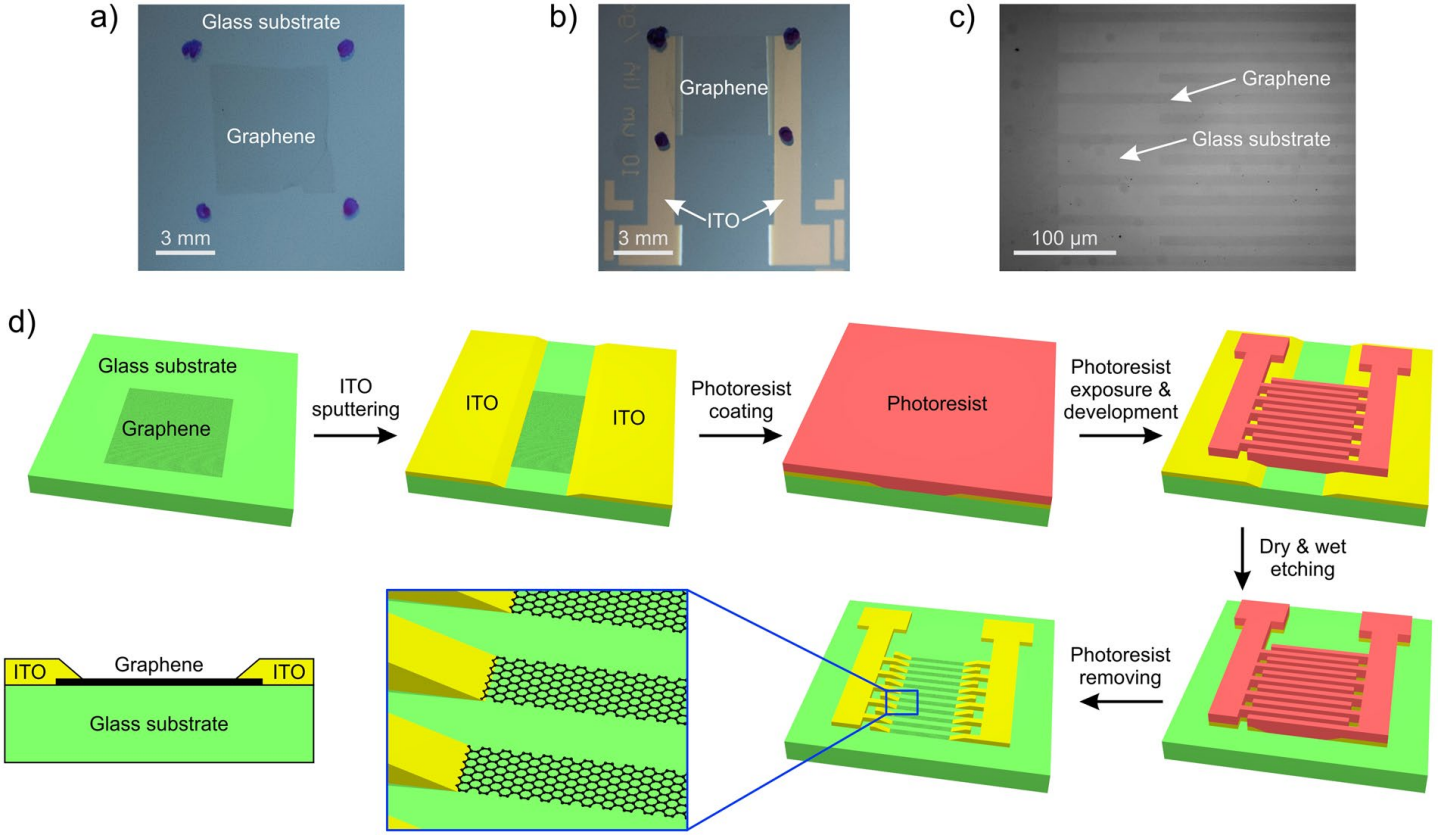
Photographs of: (**a**) glass substrate with unprocessed single layer graphene film, and (**b**) a final symmetric graphene IPS pattern on a glass substrate. (**c**) Micrograph of graphene inter-digitated electrodes at the edge IPS of pixel. (**d**) Schematic representation of fabrication steps for graphene IPS pattern with ITO contact bus lines and a cross-section sketch of the layer structure of fabricated IPS substrate.

The graphene-based LC device was fabricated from glass substrates deposited with a single layer graphene film in the center, as shown on Fig. 2(a). The electrode pattern of manufactured IPS prototype substrate consists of interdigitated graphene electrodes forming the active area of IPS pixel, and supplementary ITO contact bus lines supplying the graphene layer with driving voltage, as shown on Fig. 2(b). Due to negligibly small absorbance of graphene a backwards reflected light with optimized lighting conditions is used to demonstrate the appearance of graphene film and electrodes shown on Fig. 2(a,b and c).

In order to be able to apply electric field to the graphene layer the substrate was additionally deposited with ITO conductive layer covering the right and left edges of the graphene film. The center part of the graphene film and the substrate was protected by a shadow mask splitting the ITO layer into right and left parts which are electrically connected only through the graphene layer in the center of the substrate. Afterwards, both ITO and graphene films on the substrate were patterned using a photolithographic process with a positive photoresist and photomask to produce a single graphene IPS pixel with ITO contact bus lines, as illustrated on Fig. 2(d). The photolithography processing consisted of wet etching with acid solution for pattering the ITO film, and dry etching with plasma to pattern the graphene film. Dimensions of produced IPS pixel are 5 × 5 mm, as shown on Fig. 2(b). The electrode pattern of the IPS pixel consists of a series of 10 µm wide interdigitated electrode lines which are separated by 10 µm gaps between each two electrodes, as shown on Fig. 2(c). For a high-resolution LCD the electrode line width and gaps can be further reduced to less than 3 µm using the same photolithography process and will be only restricted by the optical resolution of the imaging system. The single pixel IPS cell is made of the IPS substrate with patterned electrodes and an uncoated cover glass substrate with no electrodes. The cell gap thickness is set by particle spacers dispersed in the glue seal and verified experimentally using the interference method to be 3.5 + /− 0.2 µm in both graphene and reference ITO cells. The ITO layer for reference ITO-based IPS prototype is deposited using the same technique as for ITO contact bus electrodes in graphene-based IPS prototype.

Durability of ITO allows to define the alignment direction of LC easily by mechanically rubbing the surface of alignment layer. However, a high sensitivity of graphene to direct mechanical impacts such as rubbing makes it nearly impossible to apply reliably industry’s standard PI alignment layer materials for a high-quality planar alignment of LC in a cell with graphene electrodes. Even a minor mechanical buffing can create substantial irreversible damage of the integrity of graphene layer. Therefore, we create a high-quality planar alignment of the LC via a non-contact photoalignment method using a commercial polymerizable photoalignment material LIA-01 (from DIC Corporation). The solution of LIA-01 contains potentially harmful to graphene NMP solvent. However, a non-contact alignment process and low temperature processing allow gentle coating of inner surfaces of both substrates successfully yielding a properly functioning IPS electrode structure. The orientation of the LC is chosen to be at 15° with respect to IPS electrodes and was optically generated in assembled empty cells producing perfectly matched alignment on both top and bottom substrates simultaneously, which is a distinct advantage of the photoalignment technique.

Characterization of manufactured IPS prototypes consisted of quantitative electro-optical measurements on optical bench, and qualitative observations between crossed polarizers and under the polarizing optical microscope (POM). The performance of graphene-based IPS prototype was evaluated by comparing its static transmittance voltage response (TV curve), response time, and contrast ratio (CR) with a reference ITO-based IPS prototype of identical design. A commercial nematic liquid crystal (NLC) mixture TMS83700 (from HCCH) having positive dielectric anisotropy (Δε = 10.6), low birefringence (Δn = 0.1), and low viscosity (γ = 0.063 Pa·s) is used in all characterization experiments with both types of manufactured IPS devices. A He:Ne laser (633 nm) is used as a light source for electro-optic measurements, and a white light for observations between crossed polarizers.

**Figure 3:**
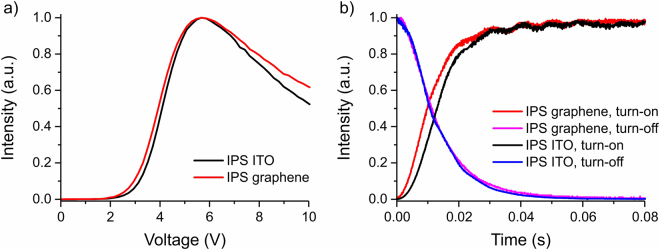
Static (**a**) and dynamic (**b**) voltage response curves of graphene IPS and reference ITO IPS prototypes.

**Figure 4:**
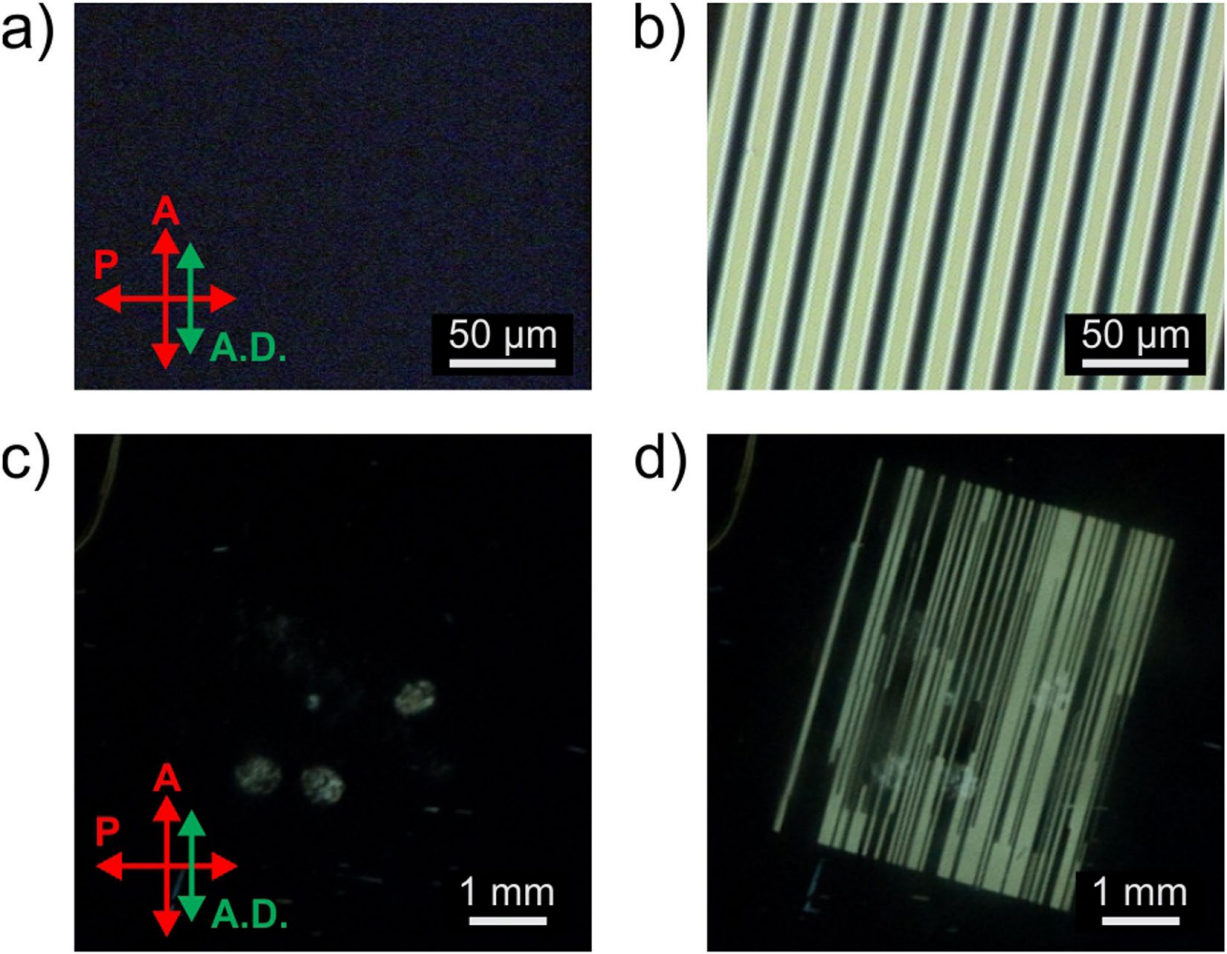
Micrographs and photographs of graphene IPS LC device between crossed polarizers at: (**a**), (**c**) 0 V; (**b**), (**d**) 5.0 V.

The TV curve of graphene IPS prototype is consistent thorough multiple switching cycles and matches well with the TV curve of the reference ITO IPS prototype. Normalized TV curves of graphene and reference ITO IPS cells measured with 1 kHz square waveform AC voltage driving match closely, as shown on Fig. 3(a). In general, smaller thickness of graphene layer can be expected to produce a minor difference between TV curves of graphene and ITO IPS devices. Thinner IPS electrodes generate weaker horizontal component of electric field in the bulk of the cell which slightly increases driving voltage, and at the same time increases the maximum brightness of LC device due to a decrease of the size of inactive areas above electrodes visible as black stripes on Fig. 4(b) ^39^. However, since the thickness of both ITO and graphene electrodes is much smaller than the cell gap thickness and dimensions of the IPS pattern this difference in driving voltage and maximum brightness is indistinguishable in current experiment. Numerical analysis confirms that visible separation between TV curves from ITO and graphene IPS prototypes has the strongest contribution from electro-optic effect of minor structural imperfections in produced IPS electrode patterns, and a weak contribution from a small deviation in the alignment orientation of LC with respect to electrodes in different cells.

The response time of both graphene, and reference ITO prototypes is measured for switching between 1 V and 5.66 V corresponding to the state of maximum transmittance, and calculated as a change of transmittance between 10% and 90%. Experimentally measured average turn-on switching time of the graphene IPS cell is 20.1 ms, which is 10.7% smaller compared to the reference ITO cell (22 5 ms). The average turn-off switching time of graphene IPS cell is 23.8 ms, and is 4.4% larger compared to the ITO IPS cell (22.8 ms). The liquid crystal surface anchoring strength on graphene substrates is comparable to that of ITO substrates manifested by the negligible difference in response times of the two cells as shown in Fig. 3(b).

The turn-off switching time of IPS LC device depends on rotational viscosity of the LC *γ*, cell gap thickness *d*, and twist elastic constant *K*_22_, and analytically can be approximated as follows^34^:

1 $${\tau }_{off(IPS)}\approx \frac{\gamma \cdot {d}^{2}}{{K}_{22}{\pi }^{2}}$$

Equation 1 with known *K*_22_ = 6.8 pN for NLC mixture TMS83700 demonstrates that observed difference in turn-off response time corresponds to a difference in the cell gap thickness of 2.2%. Empty prototype cells had a small deviation of the cell gap thickness of up to +/− 0.2 µm or +/− 5.7%. Therefore, we conclude that the response time of graphene and ITO IPS prototypes is different by no more than the accuracy of the experimental measurements.

Micrographs and photographs of the graphene IPS prototype cell between crossed polarizers are shown on Fig. 4. The photoalignment provides a high-quality and uniform alignment of the LC capable of preserving the structural integrity of graphene electrode pattern. A good area of graphene IPS cell is visually indistinguishable from a reference ITO IPS cell, and has an identical quality of the dark state. However, due to graphene’s fragility the graphene IPS prototype has notably larger amount of inactive electrodes. Such electrodes create black stripes of uneven length decreasing the maximum brightness of the graphene IPS cell, as shown in Fig. 4(d). As result, the maximum CR of the graphene IPS cell is ~130:1, compared to ~1600:1 in a reference ITO IPS cell with better photolithographic yield of the electrode pattern. At the same time, alignment defects caused by substrate contamination during small scale cell manufacturing process create undesired light leakage in a dark state, as shown in Fig. 4(c). Such defects can be observed in both graphene and ITO cells.

Inactive electrodes of graphene IPS pattern illustrate practical challenge for processing of graphene into a high-resolution electrode patterns. Narrow and long electrode lines made from graphene are more susceptible to mechanical damage during photolithography and other processing compared to more robust ITO. Current results demonstrate a successful fabrication of a high-resolution millimeter-scale IPS pattern out of monolayer graphene which is able to sustain processing required for LC devices. However, further optimization of fabrication process is required to improve the yield of photolithography and defect free alignment.

## Conclusions

In conclusion, we have demonstrated graphene-based single pixel laboratory prototype of an IPS LC device. Photolithographic patterning of single layer graphene into a high-density electrode pattern and a high-quality photoalignment allow efficient electro-optical switching of the liquid crystal. The static voltage response curve and response time of the fabricated graphene-based single pixel laboratory IPS prototype is confirmed to be equivalent to a reference ITO IPS prototype of the same design. Identical electro-optic performance and better optical transmittance of graphene compared to ITO make our findings useful for development of patterned graphene electrodes with a complex shape for displays and other liquid crystal based electro-optical devices on rigid and potentially flexible substrates.

## Methods

Graphene was grown on Cu foil (99.999%, Alfa Aesar) at 1000 °C in a low pressure (~500 mTorr) CVD furnace with CH_4_ (~50 Sccm) and H_2_ (~20 Sccm) as precursor. To transfer graphene, a thin layer of PMMA (A4, Microchem) was spin coated on Cu foil at 2000 RPM for 1 min. as a carrier layer. The backside graphene was etched by O_2_ plasma (~250 mTorr, 30 sec.). Then the film (PMMA/Graphene/Cu foil) was left in (NH_4_)_2_S_2_O_8_ (0.1 mol/L) water solution to etch Cu foil, and subsequently washed in DI water several times to clean the surface. PMMA/Graphene film was next scooped onto target quartz substrate. After the film was completely dry and tightly attached onto the substrate, the whole sample was soaked in acetone to dissolve PMMA. As a result, graphene film was transferred onto quartz substrate.

The ITO layer was deposited on the quartz glass substrates with graphene films by a pulsed DC magnetron sputtering method. Before sputtering graphene substrates were rinsed with deionized water, isopropanol (99%, BDH), and dried in air oven at 90 °C during 10 min. The reactive ITO sputtering was performed in a vacuum chamber (10^–6^ Torr) by Ar gas plasma (8 mTorr) with addition of O_2_ gas (0.44 Sccm). Magnetron power delivered to sputtering target was 500 W. The thickness of deposited ITO layer was ~10 nm and sheet resistance in the range of 125 Ω/□ − 290 Ω/□. Commercial ITO-coated soda-lime glass used in transmittance study has the thickness of ITO layer of ~25 nm and sheet resistance of 80 Ω/□. The thickness of graphene layer verified by the AFM study is not more than 4 nm, and the resistance of the plain graphene film measured through ITO contacts was in the range 4–15 kΩ, which is consistent with other literature^9^.

The ITO sputtered substrates were cleaned for a second time with isopropanol (99%, BDH), and dried in air oven at 90 °C during 10 min. Both wet and dry photolithography steps were performed with a positive photoresist S1818 (Dow Chemical). The substrates for IPS prototypes were spin coated with a diluted photoresist solution (500 RPM, 7 sec, 1500 RPM, 30 sec.) and dried on a hot plate at 95 °C during 1 min. Photoresist coated substrates with graphene and supplementary ITO layers were exposed to a UV light through a Chromium shadow mask and developed in a 2.5% water solution of tetramethylammonium hydroxide. Opened areas of photoresist pattern were exposed to an etching acid solution in order to etch the ITO layer, and later dry etched with O_2_ plasma in order to etch the graphene layer. The wet photolithography step etching was performed with water solution of 0.869 vol.% of HCl and 0.167 vol.% HNO_3_ acids during 7 min. The dry etching photolithography step was performed using O_2_ plasma (5 Sccm, 100 mTorr, 100 W, 45 sec.) in Oxford 80Plus etching machine. Substrates patterned by both etching steps were stripped from photoresist by rinsing with acetone (99%, BDH), and then were rinsed with methanol (99%, BDH), dried in air oven at 90 °C during 10 min. Both graphene and ITO substrates were coated with a commercial photoalignment material LIA-01 (DIC Corporation). Alignment layer was deposited by spin coating from a solution with ~2 wt.% of solid material (500 RPM, 7 sec, 1500 RPM, 30 sec.), and dried on a hot plate 85 °C during 3 min. The IPS prototypes cell have cell gap thickness of 3.5+/− 0.2 µm controlled by the diameter of particle spacers dispersed in an optical adhesive (NOA-68, Norland) glue seal. Assembled IPS prototype cells were exposed to linearly polarized UV light (~365 nm UV LED, 25 mW/cm^2^, 280 sec.) at a room temperature (21 °C) to generate a high-quality unidirectional planar alignment of LC molecules on the surface of both substrates. The LC is aligned normally to the polarization direction of the UV light and at 15° with respect to direction of patterned IPS electrodes.
